# Ensemble Prediction of a Halo Coronal Mass Ejection Using Heliospheric Imagers

**DOI:** 10.1029/2017SW001786

**Published:** 2018-07-02

**Authors:** T. Amerstorfer, C. Möstl, P. Hess, M. Temmer, M. L. Mays, M. A. Reiss, P. Lowrance, P.‐A. Bourdin

**Affiliations:** ^1^ Space Research Institute Austrian Academy of Sciences Graz Austria; ^2^ NRC Research Associate, U.S. Naval Research Laboratory Washington DC USA; ^3^ Institute of Physics University of Graz Graz Austria; ^4^ Heliophysics Science Division NASA Goddard Space Flight Center Greenbelt MD USA; ^5^ IPAC California Institute of Technology Pasadena CA USA

**Keywords:** space weather, coronal mass ejections, interplanetary coronal mass ejections, heliospheric imaging, prediction, ensemble modeling

## Abstract

The Solar TErrestrial RElations Observatory (STEREO) and its heliospheric imagers (HIs) have provided us the possibility to enhance our understanding of the interplanetary propagation of coronal mass ejections (CMEs). HI‐based methods are able to forecast arrival times and speeds at any target and use the advantage of tracing a CME's path of propagation up to 1 AU and beyond. In our study, we use the ELEvoHI model for CME arrival prediction together with an ensemble approach to derive uncertainties in the modeled arrival time and impact speed. The CME from 3 November 2010 is analyzed by performing 339 model runs that are compared to in situ measurements from lined‐up spacecraft MErcury Surface, Space ENvironment, GEochemistry, and Ranging and STEREO‐B. Remote data from STEREO‐B showed the CME as halo event, which is comparable to an HI observer situated at L1 and observing an Earth‐directed CME. A promising and easy approach is found by using the frequency distributions of four ELEvoHI output parameters, drag parameter, background solar wind speed, initial distance, and speed. In this case study, the most frequent values of these outputs lead to the predictions with the smallest errors. Restricting the ensemble to those runs, we are able to reduce the mean absolute arrival time error from 3.5 ± 2.6 to 1.6 ± 1.1 hr at 1 AU. Our study suggests that L1 may provide a sufficient vantage point for an Earth‐directed CME, when observed by HI, and that ensemble modeling could be a feasible approach to use ELEvoHI operationally.

## Key Points

- Heliospheric imaging at the Sun‐Earth L1 point may be supplementary to an operational space weather mission at L5
- Information on the ecliptic extent of a CME improves HI‐based predictions
- ELEvoHI ensemble prediction can be restricted by considering frequency distributions of internal fitting parameters

## 1 Introduction

Coronal mass ejections (CMEs) are the drivers of the most intense geomagnetic storms at Earth. The combination of enhanced particle density, high speed, and an enclosed magnetic flux rope with an increased magnetic field strength can lead to severe disturbances on Earth and the difficulties with predicting these phenomena are currently fueling worldwide efforts to better understand and forecast them. In the last decade NASA's twin satellites of the Solar TErrestrial RElations Observatory (STEREO) have facilitated a deep insight into the interplanetary propagation of CMEs. In particular, the wide‐angle heliospheric imagers (HIs) enabled the development of a multitude of methods for analyzing the evolution of CMEs through interplanetary (IP) space (Davies et al., 2012; Kahler & Webb, 2007; Lugaz et al., 2009; Möstl et al., 2011, 2013; Rollett et al., 2012, 2013, 2014; Rouillard et al., 2008; Sheeley et al., 1999). A recent review on HI and according methods can be found in Harrison et al. (2017).

Case studies using HI‐based prediction models assuming constant propagation speed find an arrival time error of about 8 ± 6 hr and arrival speeds are mostly overestimated by some 100 km/s (e.g., Möstl et al., 2014). In Tucker‐Hood et al. (2015) 60 CME arrival predictions were performed using the Fixed‐Phi (FPF; Rouillard et al., 2008; Sheeley et al., 1999) and Harmonic Mean fitting methods (Howard & Tappin, 2009; Lugaz et al., 2009) as well as a model including the drag force (Cargill, 2004) and a constant acceleration model (Gopalswamy et al., 2000), resulting in an absolute average error in transit time of 19 hr. In that study, STEREO HI beacon data were used, which depicts the situation of operational forecasts when using HI near‐real‐time data. Another recent study by Möstl et al. (2017) predicted the arrival of 1,337 CMEs based on HI science data from 8 years of observations and used the self‐similar expansion fitting method (Davies et al., 2012; Möstl & Davies, 2013), which assumes constant propagation speed. From this data set, 315 CMEs were detected in situ and a mean absolute arrival time error of 14.2 hr was found. It is expected that the arrival time error can be reduced when the interaction with the ambient medium is taken into account. Independent of HI data, the WSA‐ENLIL+Cone model, that is, the Wang‐Sheeley‐Arge coronal model (WSA; Arge & Pizzo, 2000; Arge et al., 2004) combined with the ENLIL solar wind model (Odstrčil, 2003), for predicting the arrival of 17 events, Mays et al. (2015) applied an ensemble approach and found a mean absolute arrival time error of 12.3 hr, which is in the same range as other studies show (Millward et al., 2013; Rollett et al., 2016; Vršnak et al., 2014). In a recent study by Wold et al. (2018), almost 7 years of operational CME arrival predictions using the WSA‐ENLIL+Cone model were assessed. During this period, 273 events were predicted and observed at Earth with a mean absolute arrival time error of 10 ± 0.9 hr. That study represents the currently achieved arrival time error when predicting CMEs at Earth as the WSA‐ENLIL+Cone model is widely used for operational space weather forecasting.

Currently, for operational forecasting mainly coronagraph observations from Large Angle and Spectrometric Coronagraph (LASCO) on board the Solar and Heliospheric Observatory (SoHO) are used. These observations have two main handicaps compared to HI observations. First, SoHO is located at the Lagrangian L1 point, situated about 1.5 million km in front of Earth along the Sun‐Earth line. This provides a head‐on vantage point of Earth‐directed CMEs, which appear as halo CMEs in such observations. The expansion of these halo CMEs is an indicator for the propagation speed (Schwenn et al., 2005) and can be used to forecast the arrival time at Earth. Second, LASCO C3 observes the space around the Sun up to 30 solar radii (R_⊙_), which corresponds to only 15% of the Sun‐Earth distance. During the earlier phase of STEREO, that is, as long as data were available from the nearside of the Sun, observations from STEREO's coronagraphs were used additionally to gain information on the CME 3‐D shape and kinematics needed for predictions (e.g., Thernisien et al., 2009). From STEREO HI observations we know that the IP propagation of CMEs is far from being undisturbed. Therefore, it is of high value to be able to follow a CME's evolution along a larger distance than coronagraphs provide (e.g., Colaninno et al., 2013).

Besides improving the prediction of arrival time and speed of a CME at Earth, there is an even more important issue, namely, to reduce the false alarm rate, which is the percentage of CMEs predicted to impact Earth that actually miss. Wold et al. (2018) indicate the false alarm rate to be 10% (with a hit rate of 50%) when predicting CME arrivals using the WSA‐ENLIL+Cone model. CMEs can be strongly influenced by different phenomena in the solar wind like other CMEs or the background solar wind itself. Besides this typical deceleration or acceleration of fast or slow events, they can be forced to change their overall direction of motion due to the influence of magnetic forces close to the Sun (Kay & Opher, 2015; Möstl et al., 2015) or due to other CMEs farther out in IP space (e.g., Lugaz et al., 2012).

The CME studied in this article erupted on 3 November 2010, associated with a C4.9 flare close to the eastern limb of the Sun peaking at 12:21 UT (Reeves & Golub, 2011). Various studies analyzed the eruption consistent with the classical standard flare‐CME model. Bain et al. (2012) studied the metric type II burst, which was associated with the eruption. The authors found that the burst was located ahead of the hot core of the erupting plasmoid, which is an indication for a piston‐driven shock. Zimovets et al. (2012) analyzed the same event in more detail and came to a similar conclusion, namely, the presence of a piston‐driven shock. They note that the shock wave may have transformed to a freely propagating blast wave during its evolution. However, Kumar and Innes (2013) discovered fast waves at the onset of the flare, which hints at the type II burst being caused by a blast wave rather than by a piston‐driven shock. Due to the exceptional good observations in extreme ultraviolet, a multitude of studies investigated the multithermal dynamics and the early stage of the eruption (e.g., Cheng et al., 2011; Foullon et al., 2011; Hannah & Kontar, 2013).

In this study, we aim to test the L1 point as a possible location for an operational HI to monitor Earth‐directed CMEs, which would be a superior supplement to a space weather mission to L5. We use the advantage of the CME on 3 November 2010, directed toward STEREO‐B and observed remotely by HI as well as in situ by the same spacecraft, to simulate the situation of an Earth‐directed CME observed from L1. Additionally, the CME was detected in situ by the MErcury Surface, Space ENvironment, GEochemistry, and Ranging (MESSENGER) spacecraft, which was almost exactly lined up with STEREO‐B during the time of the event. Ensemble predictions (339 model runs) from the current state‐of‐the‐art HI elongation fitting method ELEvoHI (Rollett et al., 2016) as well as constraining the predictions with additional information on the CME mass and on the frequency distribution of four ELEvoHI output parameters show a promising new possibility for more accurate CME arrival predictions.

## 2 Event Overview and Data

### 2.1 Remote Observations

**Figure 1:**
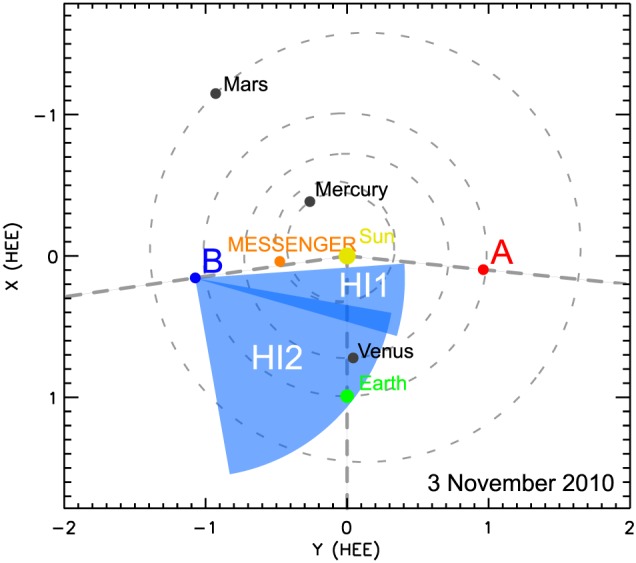
Positions of STEREO, MESSENGER, and the planets of the inner heliosphere at the time of launch of the CME under study. MESSENGER and STEREO‐B were radially aligned; both spacecraft measured the CME in situ. The fields of view of HI1‐B and HI2‐B are marked by the blue areas. For the prediction itself only HI data from HI1 are used. STEREO = Solar TErrestrial RElations Observatory; MESSENGER = MErcury Surface, Space ENvironment, GEochemistry, and Ranging; CME = coronal mass ejection; HI = heliospheric imager.

CMEs are commonly observed by coronagraphs, where the bright photospheric light is shielded by occulter disks. This enables the observation of the faint solar corona. Situated at the L1 point, the SoHO carries such instruments, LASCO C2 and C3 (Brueckner et al., 1995), having a field of view of 2 to 6 R_⊙_ and 3.7 to 30 R_⊙_, respectively. The STEREO mission was launched in 2006 and consists of twin satellites, STEREO‐A(head) and STEREO‐B(ehind), both equipped with the same set of instruments. Part of STEREO's SECCHI suite (Howard et al., 2008) are two coronagraphs, COR1 and COR2, observing an area of 1.4 to 4 R_⊙_ and 2 to 15 R_⊙_ around the Sun. At the time of the CME event under study, STEREO‐A was 84° ahead of Earth, STEREO‐B was 82° behind, that is, they were separated by 166°. In addition to coronagraph observations, we use data from the HIs, HI1 and HI2, ecliptic centered wide‐angle white light cameras observing the space between the Sun and 1 AU and beyond. HI1 has an angular field of view of 4° to 24° elongation (the angle between the Sun‐spacecraft line and the line of sight), HI2 observes from 18° to 88° elongation. Figure 1 shows an ecliptic cut of the positions of STEREO, MESSENGER, and the planets of the inner heliosphere. The blue shaded areas mark the fields of view of HI1‐B and HI2‐B. In this study, only HI data from HI1‐B are used for the ELEvoHI arrival predictions; HI2‐B data are used to prove the consistency of predicted CME evolution and observations.

The CME under study was first observed by LASCO C2 on 3 November 2010 at 12:36 UT and entered the field of view of C3 at 14:06 UT. In STEREO‐B COR2 the CME was visible as a halo, while in STEREO‐A COR2 it appeared as backside halo CME. It entered the field of view of STEREO‐B HI1 on 4 November 2010 at 4:49 UT and the STEREO‐A HI1 field of view at 3:29 UT. In STEREO‐A and STEREO‐B HI2 the CME was first visible on 5 November 2010 at 10:10 UT.

### 2.2 In Situ Observations

**Figure 2:**
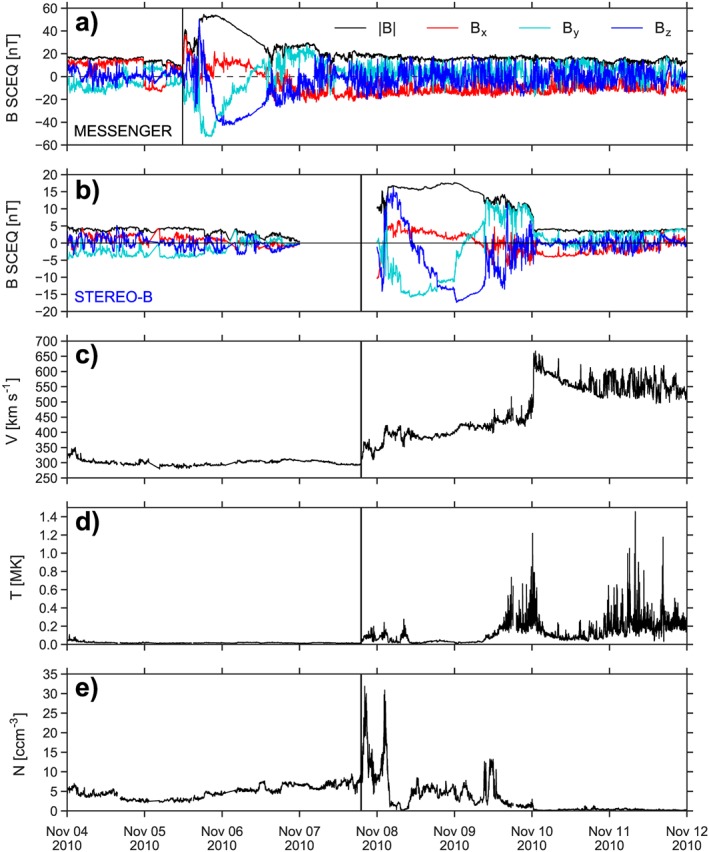
In situ observations by MESSENGER and STEREO‐B of the early November 2010 interplanetary coronal mass ejection. Panel (a) shows the magnetic field components (B_x_ red, B_y_ turquoise, and B_z_ blue) and total field (black) at MESSENGER in the SpaceCraft Equatorial Coordinate system, which is similar to Heliocentric Earth Equatorial coordinates except that the system is centered on the spacecraft, not Earth. The vertical solid lines indicate the arrival of a shock. Panel (b) shows the magnetic field at STEREO‐B in a similar format. The proton bulk speed at STEREO‐B is shown in panel (c); panel (d) displays the proton temperature and (e) the density. The shock arrival time at STEREO‐B at the solid vertical line is derived from the plasma parameters as the magnetic field has a data gap. STEREO = Solar TErrestrial RElations Observatory; MESSENGER = MErcury Surface, Space ENvironment, GEochemistry, and Ranging; SCEQ = SpaceCraft Equatorial Coordinates.

The first detection of the CME shock by the MESSENGER spacecraft was recorded on 5 November 2010 at 11:46 UT, while MESSENGER was situated at 0.48 AU 84° east of Earth. During its cruise phase between August 2004 and March 2011, the magnetometer on board MESSENGER (Anderson et al., 2007) measured the IP magnetic field vector in the solar wind. Figure 2a shows the magnetic field vector in SpaceCraft Equatorial Coordinates with red, turquoise, and blue lines being the x, y, z components and the black line being the total magnetic field. In the SpaceCraft Equatorial Coordinates coordinate system, the z axis is the solar rotation axis, the x axis points from the Sun to the spacecraft, and y completes the right‐handed triad, pointing to solar west. The flux rope passed over the spacecraft between 16:53 and 13:24 UT, having a right‐handed chirality and an axis orientation with a low inclination relative to the ecliptic plane (Bothmer & Schwenn, 1998).

At 7 November at 19:05 UT the CME shock arrival was detected by STEREO‐B, located at 1.08 AU and 82° east of Earth. In contrast to MESSENGER, STEREO also provides plasma measurements. The CME sheath region arrives with a speed of ≈350 km/s, while during the flux rope interval, the speed is ≈400 km/s during its first half and increases to more than 450 km/s (Figures 2b and 2c). The reason for this speed increase seems to be the high‐speed solar wind stream, which is pushing the magnetic flux rope from behind. The result of this interaction is a reverse shock behind the flux rope with a speed of ≈600 km/s. The magnetic flux rope started on 8 November at 03:28 UT and lasted until 9 November at 09:11 UT. Similar to the magnetic signature at MESSENGER, we find a low inclined flux rope with a positive chirality, so the overall flux rope structure has not changed. Usually, CMEs expand during their IP propagation, which increases their duration and decreases their magnetic field strength. This event is no exception, as the mean magnetic field strength in the magnetic flux rope has decreased by a factor of 2.7 from 43.6 ± 8.6 nT at MESSENGER to 16.2 ± 0.9 nT at STEREO‐B. The duration of the flux rope at STEREO‐B (30.3 hr) is 50 % larger than the duration at MESSENGER (20.2 hr).

During the CME impact the Spitzer Space Telescope was located about 34° west of STEREO‐B, which provides an opportunity to further test ELEvoHI with an additional in situ detection from a third spacecraft. A good indication for space weather events at Spitzer is the number soft scrub errors, which are single‐bit errors at any memory location. These soft scrub errors can be directly related to solar flares or CMEs (Cheng et al., 2014), but no increase in these errors was observed. Since Spitzer is mostly affected by high‐energy particle hits, that is, energies of about 100 MeV and from the High Energy Telescopes from STEREO‐B/IMPACT (Luhmann et al., 2008; von Rosenvinge et al., 2008), we conclude that no high energetic particles have been observed during the time of arrival of the CME.

## 3 Methods

### 3.1 Graduated Cylindrical Shell Fitting

In order to conduct a CME arrival prediction using ELEvoHI, we need information on the shape of the CME within the ecliptic as input. To determine the CME geometry in the corona, the Graduated Cylindrical Shell (GCS) forward modeling technique (Thernisien et al., 2006, 2009) is employed. This model reduces the CME magnetic ejecta, that is, the flux rope, to a function of six free parameters: the propagation longitude and latitude, the tilt angle of the CME central axis, the separation width of the CME legs, the aspect ratio between the CME major and minor cross sections, and the height of the CME nose at a particular time. These parameters are determined by utilizing approximately cotemporal images from SECCHI and LASCO to fit the proscribed geometry to what is observed from multiple viewpoints at different times. As in Hess and Zhang (2015), most parameters are kept as fixed as possible to provide a unique solution to the CME geometry. However, while that study focused only on fast CMEs, the slower speed of the 3 November 2010 CME required a slight adjustment to the longitude with time to account for solar rotation.

GCS fitting to this CME yields a best fit latitude of −2°, a tilt angle of 17°, a half angle of 39°, and an aspect ratio of 0.3. These parameters were fixed throughout the propagation. The Carrington longitude was gradually changed from 219° to 209°. The height of the nose was 13.43 R_⊙_ at 00:54 UT on 4 November and the last measurement performed in HI1 had a height of 93.28 R_⊙_ at 06:09 UT on the 5 November. Observationally, there appears to be a coherent flux rope structure in the coronagraph data that serves as the basis for these fits. When processing the data with a running difference, another structure is visible, which can be a sign of a CME‐driven shock (Hess & Zhang, 2014). Regardless of the source of this structure, it did provide a complication in determining the exact extent of the CME width. In order to try and get a sense of the possible error of the event, an extremely wide fit was performed to include this structure. Most of the parameters are similar to the original fit performed, but the aspect ratio was increased to 0.36 and the half‐angle width was 58°. This CME is almost certainly too wide, but it may be a better fit to the entire density structure that is visible, especially in HI1.

**Figure 3:**
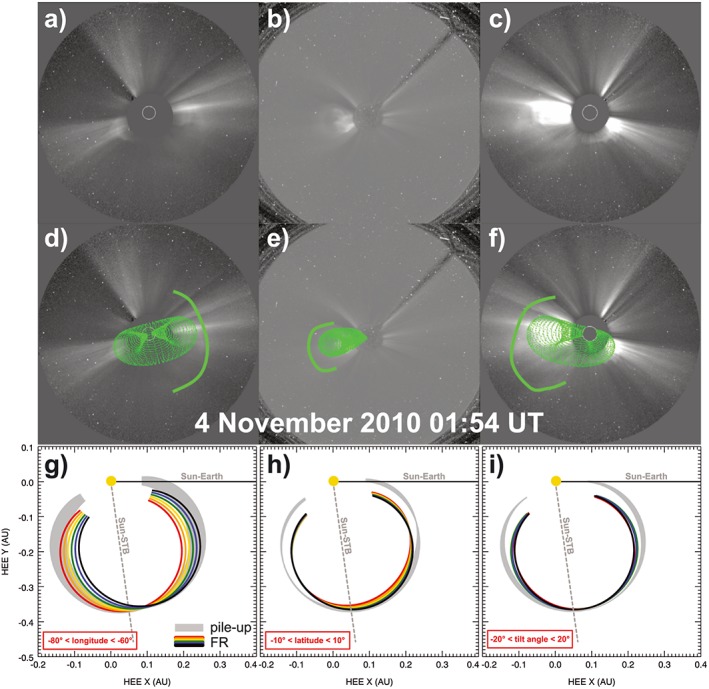
Base difference images of COR2‐B (a, d), Large Angle and Spectrometric Coronagraph/C3 (b, e), and COR2‐A (c, f) observations. Panels (d)–(f) show the GCS shape fitted to what is believed to be the ejecta. The outline is to highlight another structure present in the data, which was used to determine the maximum possible extent of the eruption and might be part of a shock front. Panels (g)–(i) show the intersection of the GCS shape with the ecliptic to determine the input parameters needed for ELEvoHI. The colored ellipses show the shape variations of the eclitpic cut when varying the tilt angle (g), the longitude (h), and the latitude (i) within their error ranges; the gray area shows the variation of the ecliptic cut of the GCS fit to the CME density pileup. GCS = Graduated Cylindrical Shell; CME = coronal mass ejection.

The GCS model provides a three‐dimensional geometry in the corona. To generate the inputs for ELEvoHI, the extent of the CME leading edge in the ecliptic plane must be determined. As first presented by Colaninno et al. (2013), this can be done analytically utilizing the detailed geometry of the model presented in Thernisien (2011). If the CME is propagating well away from the ecliptic or has a significant tilt, the ecliptic cut of the GCS geometry will vary more significantly due to slight changes. However, for a CME with a central axis that is close to the ecliptic plane, this will be less significant. For further investigation, we take into account an error range of the GCS parameters of −80° ≤ longitude ≤−60°, −10° ≤ latitude ≤+10°, and −20° ≤ tilt angle ≤+20°. As we use only the outcome of the GCS model, which is related to the shape of the CME front, other parameters from GCS fitting are not taken into account. Panels (d)–(f) of Figure 3 show the GCS shape overlaid on base difference images (panels a–c) produced from observations of COR2‐B, LASCO C3, and COR2‐A. The green line marks the boarder of the possible shock front fitted to determine the maximum extent of the CME. The lowest panels of Figure 3 show the variation of the intersection of the GCS shape with the ecliptic, that is, the ecliptic cut, when the GCS longitude (g), the latitude (h), or the tilt angle (i) are varied within estimated errors of the GCS model leading to the possible range of the ELEvoHI input parameters. The gray areas mark the attempt to fit the GCS model rather to the dense area surrounding the ejecta. This approach might be more consistent with other assumptions of ELEvoHI, especially when tracking a CME in HI at its shock front and not at its cavity that corresponds to the flux rope (e.g., Lugaz et al., 2005).

### 3.2 ELEvoHI

ELEvoHI is a CME prediction utility first presented in Rollett et al. (2016), where it was applied to 21 CMEs observed by HI. It uses HI observations as input and predicts arrival times and speeds at the target of interest. ELEvoHI combines several methods, which are briefly described below: the Ellipse Conversion method (ELCon; Rollett et al., 2016), drag‐based model fitting (DBMF; Rollett et al., 2016), and the Ellipse Evolution model (ElEvo; Möstl et al., 2015), which is based on the drag‐based model (DBM; Vršnak et al., 2013).

The first part of this prediction tool is the ELCon method (Rollett et al., 2016), which converts the observed HI elongation angle into a unit of distance; that is, it reveals the IP CME kinematics. In this study, the time‐elongation profile was manually extracted from running difference HI images. Another possibility and maybe more feasible for real‐time predictions is to use time‐elongation maps (Davies et al., 2009), where the evolution of a CME is visible as a bright track. For the conversion of HI data, ELCon needs information about the shape of the CME within the ecliptic. This information can be gained from either coronagraph observations as in this study (see section 3.1) or average values for CME width and ellipse aspect ratio can be used, while the CME propagation direction could be found by using the FPF, HMF, or self‐similar expansion fitting method HI elongation fitting methods (cf. Rollett et al., 2016). After the application of ELCon, the CME kinematics are obtained, and the initial time, t_init_, further needed for DBMF, needs to be set. In this study, we fixed t_init_ to the second measurement point in the HI time‐elongation profile, which corresponds to t_init_= 4 November 2010 07:53 UT and an elongation value of 5.14°. After conversion from elongation to distance using ELCon and after the time derivation of the resulting time‐distance profile to gain the speed profile, we find r_init_ and v_init_ corresponding to t_init_. The mean initial values for the whole ensemble are r_init_=32 ± 2.8 R_⊙_ and v_init_=541 ± 42 km/s. ELCon provides the possibility to modify the extent of the CME shape within the ecliptic plane as suitable for each event under study. Depending on the geometry of the run (as we vary the shape for each of the 339 predictions), r_init_ varies between 26 and 40 R_⊙_ and v_init_ varies between 460 and 660 km/s. Besides the direction of motion, ϕ, the aspect ratio of the ellipse semiaxes, f, and the angular half width, λ, can be fixed, each of the three parameters is assumed to stay constant during propagation. For the equations used by ELCon, we refer to the appendix section in Rollett et al. (2016).

The next technique implemented within ELEvoHI is DBMF, numerical fitting (downhill simplex method) of the ELCon time‐distance profile using a drag‐based equation of motion (Vršnak et al., 2013). Here it is important to note that although DBMF is based on the same equations as DBM it is used in a different way. In DBMF, a time‐distance profile is numerically fitted, while the DBM is a prediction tool, which propagates a CME based on several input parameters without using information on the time evolution of the CME. In both methods it is assumed that the propagation of a CME is exclusively dominated by the drag force exerted by the solar wind:

(1) $$R_{\text{DBM}}(t)=\pm \frac{1}{\gamma }\ln[1\pm \gamma (v_{\text{init}}-w)t]+\mathrm{wt}+r_{\text{init}},$$

where R_DBM_(t) is the radial distance from Sun‐center in R_⊙_, γ is the drag parameter, which is usually ranging between 0.2 × 10^−7^ and 2 × 10^−7^ km^−1^. v_init_ and r_init_ are the initial speed and distance, respectively, and w is the background solar wind speed. The drag parameter is defined as

(2) $$\gamma =c_{D}\frac{A\rho_{\text{sw}}}{m_{\text{CME}}},$$

with c_D_ being a dimensionless drag coefficient (assumed to be 1), A is the CME cross section the drag is acting on, ρ_sw_ is the solar wind density, and m_CME_ is the CME mass. r_init_ as well as the end point of the fit usually need to be defined manually. The ELCon time‐distance profile is fitted between ≈ 30 and 100 R_⊙_, while the distance between STEREO‐B and the Sun is 233 R_⊙_. The sign ± is positive when v_init_>w and negative when v_init_<w. To find the most adequate value for w, ELEvoHI reads in in situ data from 1 AU from the same time range as the HI observations and performs several fits with different values for w, depending on the mean and extreme values of the solar wind data during this time range. The fit with the smallest residuals reveals an estimate of the average background solar wind speed, which is further used in the model. We note that this approach is suitable for real‐time prediction since both kinds of data (HI and in situ solar wind speed from 1 AU) are available in (near) real time. Another approach of DBMF is presented by Žic et al. (2015), who iteratively fit a time‐distance profile using successive input from HI. The last step of ELEvoHI is to perform the prediction. This is done by the Ellipse Evolution model (ElEvo; Möstl et al., 2015), which uses the information gained by ELCon and DBMF as input. ElEvo assumes the same shape as used for ELCon and runs the DBM (Vršnak et al., 2013) to perform the prediction.

### 3.3 CME Mass Determination

The IP propagation of a CME is assumed to be mainly dominated by the interaction between the ambient solar wind and the CME. Here the CME mass plays a major role. Since one of the outputs of ELEvoHI is the drag parameter (see equation (2)), which is a function of the CME mass, it is our intent to verify this result by comparing the CME mass calculated from the ELEvoHI outcome to the mass derived from coronagraph observations.

**Figure 4:**
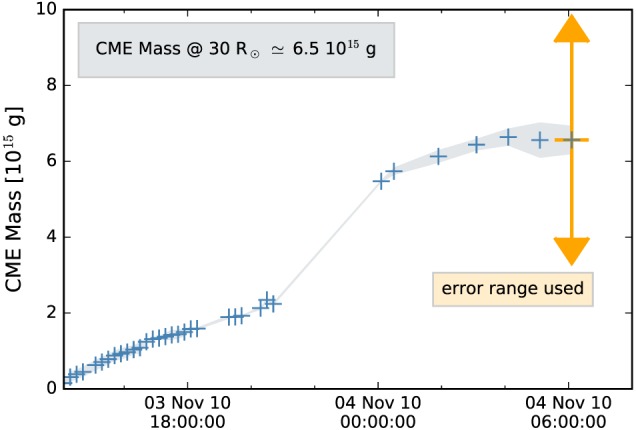
CME mass evolution versus time derived from Large Angle and Spectrometric Coronagraph C3 white light imagery covering the distance range ∼5–30 R_⊙_. The final mass is derived as average over the last three data points, between 4 and 6 UT on 4 November 2010. The orange error bar marks the POS mass range, which is further used in this study. CME = coronal mass ejection.

CMEs can be observed in white light as photons are scattered off the coronal electrons which build the CME structure. Assuming that the CME lies in the plane‐of‐sky (POS), we derive the CME mass evolution using the excess brightness as measured from white light data. LASCO C3 data preparation was done to correct for instrumental effects and calibrate in units of mean solar brightness. To derive the excess brightness a preevent image is subtracted (see, e.g., Vourlidas et al., 2000). Assuming that the ejected CME material consists of completely ionized hydrogen (90%H) and helium (10%He) the mass is calculated using the Thomson scattering function by Billings (1966). As shown in Figure 4, the CME mass evolves very slowly over several hours, before a strong increase is observed. This can be interpreted as a slow streamer‐blowout CME. Since we describe in the beginning the type II burst related to the CME and estimated speeds of the order of 1,500 km/s, this might need some additional explanation: Though the CME started very impulsively and produced a type II burst (e.g., Bain et al., 2012), the further evolution is rather moderate and the POS speed over LASCO field of view yields about 250 km/s. The rapid deceleration, deviation from radial propagation, and slow increase in mass would suggest that the CME might have interacted with a streamer, resulting in its blowout (e.g., Eselevich et al., 2015). However, here we have to note that the initial speed at ≈30 R_⊙_ derived from HI observations lies in a range of 490–570 km/s, which seems to be more reliable than the speed derived from coronagraph observations as the CME arrived with 350–400 km/s at 1 AU. The final mass for the fully developed CME, as observed in LASCO/C3 close to the outermost boundary of C3 FoV at 30 R_⊙_, is derived over the time range from 4 to 6 UT on 4 November 2010 (last three data points in Figure 4) with m_30_≈6.5 × 10^15^±0.5 × 10^15^ g.

Although the CME under study was a limb CME from the vantage point of LASCO, the derivation of the POS mass might be defective. Colaninno and Vourlidas (2009) showed that using LASCO observations leads to a lower CME mass than using STEREO observations. In a recent study, de Koning (2017) compared the POS mass (derived from one viewpoint, similar to this study) and the deprojected mass (two viewpoints) to the CME mass calculated from three separate points of view of eight CMEs. That study demonstrates the high uncertainty in the derivation of the CME POS mass of up to ±50%. In order to take into account this error range, we assume for further analysis the POS mass lying in a range between 3.25 × 10^15^ and 9.75 × 10^15^ g, which is marked in Figure 4 by the orange error bar.

## 4 Ensemble of ELEvoHI predictions

### 4.1 Determine the CME Shape and Direction

In Rollett et al. (2016) ELEvoHI used the FPF elongation fitting method to determine the direction of motion of the CME. In that study, for all of the 21 predicted CMEs the same angular half width and ellipse aspect ratio was used. In this study, we aim to optimize ELEvoHI by using a frontal shape derived from the GCS model applied to additional coronagraph data. Using the GCS model, it is possible to gain not only the propagation direction but also the angular half width and the ellipse aspect ratio by performing an intersection of the GCS shape with the ecliptic plane.

From the cut of the GCS fit with the ecliptic plane we measure the input parameters for the CME shape needed by ELEvoHI, that is, the propagation direction, *ϕ*, the inverse ellipse aspect ratio of the semiaxes, *f* = *b*/*a*, and the angular half width, *λ*. A full examination of the errors in the GCS model has not been undertaken, but based on experience with the model and the cross comparison of fits between various individuals, we believe these values are reasonable and conservative. Additionally, as we are performing an ensemble prediction, the input parameters related to the outcome of the GCS model are varied in a wide range being much larger than any error estimated. A change in the longitude is the most obvious in the ecliptic cut for the 3 November 2010 CME as it controls the pointing of the nose of the CME. Because this CME is low tilt and from a near‐equatorial latitude (in coronagraph observations), varying those parameters has very little effect on the shape of the CME. Even the effect of the longitude is not likely to affect the results near the CME nose but may impact the ability to determine the exact extent of the longitudes that will or will not be impacted by the CME flank and therefore may be a source of both missed detections and false alarms for CMEs that propagate farther from the Sun‐Earth line.

Taking into account the variations of the GCS fit to the dense CME parts as well as to the ejecta, we find the following range of the ELEvoHI input parameters: 2° ≤*ϕ*≤14°, 0.76≤*f*≤1, and 55° ≤*λ*≤85°, having steps of Δ*ϕ* = 2°, Δ*f* = 0.04, and Δ*λ* = 5°. Within these boundaries we perform *N* = 343 runs for the input triplets {*ϕ*,*f*,*λ*} with *n*_*ϕ*_=7, *n*_*f*_=7, and *n*_*λ*_=7, that is, every possible combination of the three input parameters is part of the ensemble. For the triplets {12°, 0.76, 85°}, {14°, 0.76, 85°}, {14°, 0.76, 75°}, {14°, 0.76, 80°} no solutions exist; that is, DBMF does not converge and the total number of runs reduces to *N* = 339.

### 4.2 ELEvoHI Forecast

**Figure 5:**
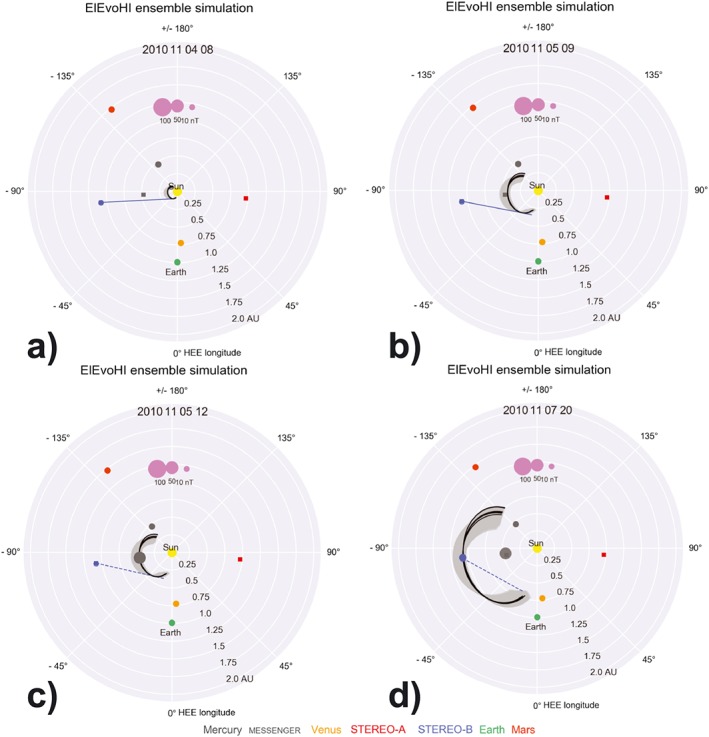
Visualization of ELEvoHI results. The black curves correspond to the CME shapes leading to the best prediction at MESSENGER and STEREO‐B, respectively. The dark gray area is the entity of all other runs from the ensemble. In panels (a) and (b) the blue tangent corresponds to the first and last elongation measurement used for the prediction. These elongations correspond to the times, where the CME apex is between 30 and 100 R_⊙_, that is, the start and end times of the fit. In panel (c) the CME arrives at MESSENGER; the dashed tangent proofs the consistency with HI observations, which are not taken into account for the predictions anymore. The size of the filled circle at the location of MESSENGER marks the magnetic field strength measured in situ. In panel (d) the CME impacts STEREO‐B; the dashed line marks the last HI observation. An animated version of this figure is available in the supporting information. CME = coronal mass ejection; STEREO = Solar TErrestrial RElations Observatory; MESSENGER = MErcury Surface, Space ENvironment, GEochemistry, and Ranging; HI = heliospheric imager.

Figure 5 shows four different time steps of the ELEvoHI ensemble prediction. In each panel, the Sun is in the center, MESSENGER is marked as a gray square, STEREO‐B is marked as a blue filled circle. Panel (a) shows the time of the first HI elongation measurement (blue tangent) used as input, panel (b) the time of the last HI elongation measurement used for the predictions. The black ellipses correspond to those runs with an arrival time within ±0.5 hr at MESSENGER and STEREO‐B, respectively. The dark gray area is the whole ensemble. Panel (c) shows the time of the in situ arrival at MESSENGER; the dashed tangent shows the HI elongation measurement from the same time, still consistent with the model output, but not used for prediction anymore. Panel (d) shows the CME impact at STEREO‐B; the dashed blue line marks the last elongation measurement. An animated version of Figure 5 is available in the supporting information.

From the 339 predictions performed, 50 lead to an arrival time error of less than ±1 hr at MESSENGER as well as at STEREO‐B. Reducing the arrival time window to ±0.5 h results in 22 events. Eighty‐three percent of the predictions lie within ±6 hr. The best arrival time prediction yield the triplets {2°, 0.8, 80°} and {10°, 0.92, 80°} with −2 min at MESSENGER and −2 min at STEREO‐B. Negative values mean that ELEvoHI predicts the arrival earlier than observed. The ensemble mean of the prediction at MESSENGER is Δ*t* =− 0.6 ± 2.7 hr; the ensemble mean at STEREO‐B is Δ*t* =− 0.9 ± 4.2 hr. The mean absolute error at MESSENGER is Δ*t* = 2.2 ± 1.6 hr and Δ*t* = 3.5 ± 2.6 hr at STEREO‐B. The ensemble median is −0.21 hr at MESSENGER and −0.03 hr at STEREO‐B. The mean predicted arrival speed is 484 ± 23 km/s at MESSENGER and 438 ± 11 km/s at STEREO‐B, while the in situ data show a speed variation in the sheath region between 350 and 400 km/s.

### 4.3 Sensitivity Analysis

**Figure 6:**
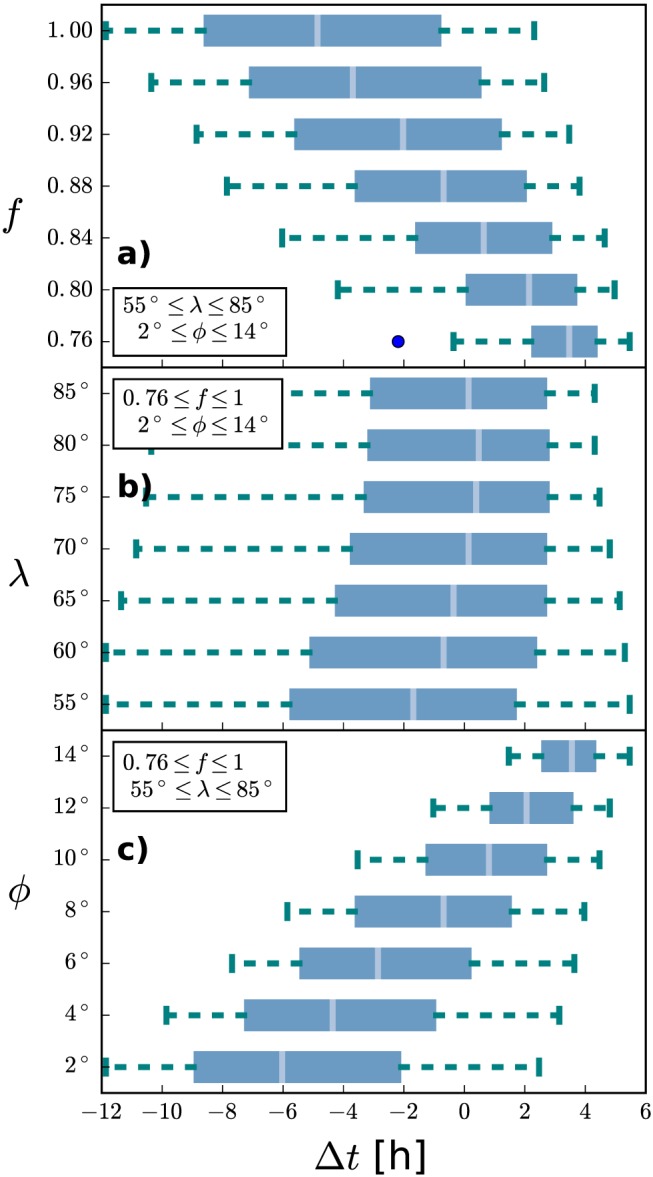
Box and whiskers plots of Δt for runs with one input parameter being fixed and the other two varying within their error ranges for each value of f, λ, and ϕ (a–c). The boxes encompass 50% of the data, with the vertical gray line representing the median. The whiskers extend out to 1.5 times the interquartile range. The blue point in panel (a) marks an outlier.

In order to test the robustness of ELEvoHI as a function of its shape‐related input parameters, we examine the influence of each of the three input parameters by an analysis of the prediction variance. For each of the seven different values of each input parameter, the runs are arranged into groups. Box and whiskers plots for all groups for the different values of *f* (*λ*, *ϕ*) are displayed in Figure 6a (b, c). The *x* axis shows the time difference between the predicted and observed arrival times, meaning that positive values correspond to an overestimated transit time. The gray vertical lines mark the medians, the boxes encompass 50% of the data, and the whiskers extend out to 1.5 times the interquartile range. The variance of the medians for the grouped results corresponding to fixed values of *f* is *σ* ^2^=7.9 hr (*σ* = 2.8 h), while the median of all medians is −0.7 hr. For the fixed values of *λ* we find a variance of the medians of *σ* ^2^=0.5 hr (*σ* = 0.7 hr), while the median of all medians is 0.1 hr and for the fixed values of *ϕ* the variance of the medians is *σ* ^2^=10.5 hr (*σ* = 3.2 hr), and the median of all medians is −0.7 hr. In this case study, the highest influence on the prediction result has the direction of motion, meaning that a change of *ϕ* of 12° leads to a difference in arrival time of ≈10 hr. In contrast, if an angular half width of *λ* = 55° or *λ* = 85° is used, only leads to a difference of 0.5 hr. However, it is important to note that this CME is propagating approximately toward STEREO‐B, which minimizes the influence of the CME shape on the prediction result. This may be different for events where not the CME apex is hitting the target of interest, and it is likely to be of high importance when predicting flank encounters, where the CME width is a decisive factor if an impact is predicted or not.

## 5 Limiting the Ensemble Results

An ensemble prediction is a great possibility to reveal the range of feasible prediction results and their occurrence frequencies. But are the most frequent predictions also the most accurate predictions? Is the mean or median value of the ensemble prediction a proper candidate to be used as resulting prediction? Is it possible to pin down the ensemble results to a most likely one? In this section, we explore the ensemble results to find a way to narrow down the forecasting range based on the CME mass or the occurrence frequencies of four resulting parameters, that is, the drag parameter, the background solar wind speed, and the initial distance and speed.

### 5.1 Limitation Using the CME Mass

In order to relate the CME mass derived from coronagraph observations to the ELEvoHI results, we now calculate the mass from ELEvoHI results using the definition of *γ* from equation (2) and rearrange

(3) $$m_{\text{CME}}=c_{D}\frac{A\rho_{\text{sw}}}{\gamma }.$$

The cross section, *A*, is calculated assuming an ellipse perpendicular to the ecliptic plane, with the same semimajor axis, *a*, as resulting from ELCon. The semiminor axis was calculated based on the angular width perpendicular to the ecliptic plane measured from the GCS fits, being 17° for the flux rope GCS fit and 21° for the GCS fit to the CME density pileup. The solar wind mass density, *ρ*_sw_, was calculated using the density model by Leblanc et al. (1998), being simply a function of solar radial distance.

**Figure 7:**
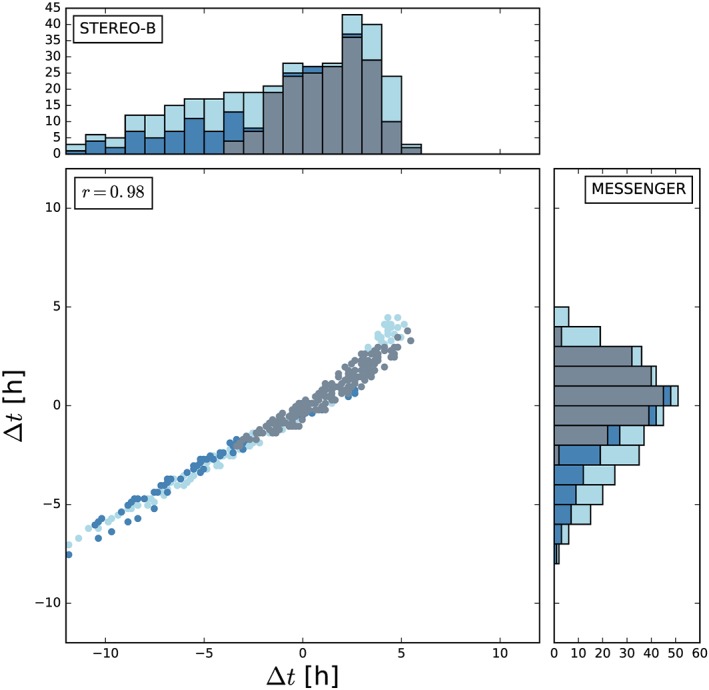
Histogram of differences of observed and predicted arrival times, Δt, at STEREO‐B (top) and MESSENGER (right). Positive values mean that the transit time is overestimated by ELEvoHI. The light blue bars show the distribution of all 339 runs, while the blue (gray) bars mark the runs with the calculated mass based on the wide GCS fit to the coronal mass ejection shock (narrow GCS fit to the flux rope) lying in a range of ±50% of the mass calculated from coronagraph images. The correlation between Δt at MESSENGER and STEREO‐B is shown in the middle plot and yields a correlation coefficient of r = 0.98. STEREO = Solar TErrestrial RElations Observatory; MESSENGER = MErcury Surface, Space ENvironment, GEochemistry, and Ranging; GCS = Graduated Cylindrical Shell.

The CME mass was calculated for each run at *r*_init_, located at 32 ± 2.8 R_⊙_ on average. The CME mass derived from coronagraph observations at 30 R_⊙_ (*m*_30_=6.5 × 10^15^±3.5 × 10^15^ g) is now used to verify parts of the ELEvoHI ensemble run. From the whole ELEvoHI ensemble, we find a median CME mass of 4.8 × 10^15^ g with 50% of the runs resulting in a mass between 3.4 × 10^15^ and 6.4 × 10^15^ g using an angular width of 21°. Using an angular width of 17° we find a median mass of 3.9 × 10^15^ g with an interquartile range between 2.7 × 10^15^ and 5.1 × 10^15^ g. However, this calculation is only a rough approximation because the whole approach is highly sensitive to the angular width perpendicular to the ecliptic. Figure 7 shows a histogram of the differences between observed and predicted arrival times at STEREO‐B (top panel) and MESSENGER (right panel) for the whole ensemble (light blue bars). The dark blue bars show the number of runs (111 events, i.e., 33%), for which the masses derived from ELEvoHI based on the GCS fit to the CME density pileup lie within ±50% of the mass calculated from coronagraph images, that is, within the assumed error, the gray bars represent the same based on the GCS flux rope fit (156 events, i.e., 46%). The mean arrival time difference of the sample based on the CME density pileup is −0.6 ± 3.7 hr (median 0.14 hr) at STEREO‐B (compared to Δ*t* =− 0.9 ± 4.2 hr for the whole ensemble) and 0.4 ± 2.2 hr (median −0.2 hr) at MESSENGER (compared to Δ*t* =− 0.6 ± 2.7 hr). The mean arrival time difference of the sample based on the CME flux rope is 1.3 ± 2 hr (median 1.5 hr) at STEREO‐B and 0.7 ± 1.3 hr (median 0.8 hr) at MESSENGER.

From the constraint using the CME mass based on the GCS fit to the CME shock we find no significant improvement for the arrival prediction. The error range is only reduced by about half an hour. In case of the constraint using the ELEvoHI mass derived from the more narrow angular width revealed by GCS fitting, the error range could be reduced by almost 50%. However, the mean and especially the median values of the constraint based on the GCS fit to the CME flux rope are shifted to a later arrival time, while the constraint based on the GCS shock fit is centered around zero. From the comparison of the ELEvoHI mass to the POS mass derived from coronagraph observations, we find that both methods reveal the CME mass in the same order of magnitude, which verifies the drag‐parameter resulting from DBMF to a certain degree. Here we have to note that the calculation of the CME mass, be it using ELEvoHI or using coronagraph images from only one vantage point (de Koning, 2017), is highly defective and sensitive to small changes of, for example, the angular width. Therefore, this approach can only be seen as a rough approximation.

The scatter plot in Figure 7 shows the correlation between Δ*t* at STEREO‐B and MESSENGER for the whole ensemble (light blue) and for the mass‐constrained predictions (dark blue and gray). We find a correlation coefficient of *r* = 0.98, meaning that an ELEvoHI run leading to a good prediction at ≈0.4 AU also leads to a good prediction at 1 AU. This is not proven for events that hit the spacecraft with its flank or for not completely aligned spacecraft. Additionally, the correlation could be different if the CME has a higher speed and a higher drag parameter than the event under study or if the CME frontal shape is not in agreement with the elliptic assumption of ELEvoHI. However, there already are studies testing the ability of spacecraft located closer to the Sun along the Sun‐Earth line to improve predictions, especially the prediction of the *B*_*z*_ component of the magnetic flux rope within the CME (e.g., Kubicka et al., 2016).

### 5.2 Limitation Using γ, w, r_init_, and v_init_

**Figure 8:**
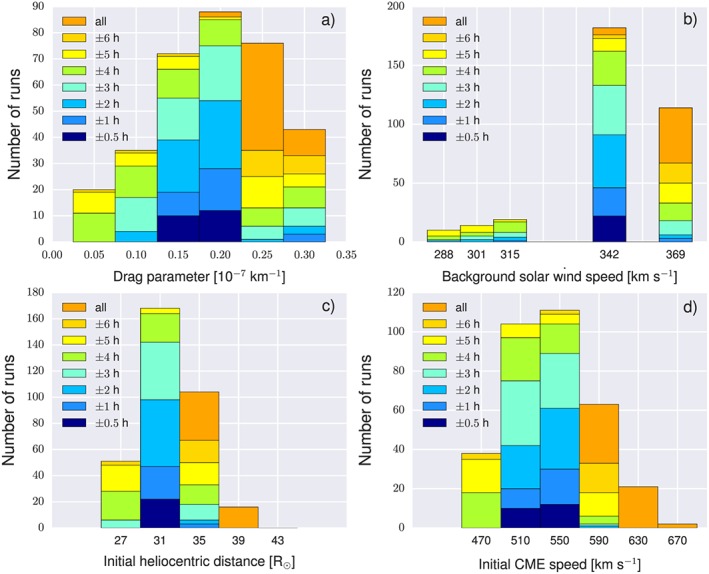
Frequency distribution of resulting drag parameter (a), γ, and background solar wind speed (b), w, initial heliocentric distance (c), and initial speed (d) resulting from all ELEvoHI runs and color coded based on the predicted and observed arrival time differences.

ELEvoHI results cover the drag parameter, *γ*, and the background solar wind speed, *w*, both obtained by DBMF applied to the ELCon time‐distance profile. As described in section 3.2, the in situ solar wind speed from 1 AU from the same time range as the HI observations is used to find the best candidate of *w* for the fit. Five different fits are performed for five different values of *w* within the minimum and the maximum values of the in situ solar wind speed. The fit with the smallest residuals reveals the resulting *w*. More information on this procedure can be found in Rollett et al. (2016). In contrast to *w*, *γ* is in fact a true fitting result of DBMF. Furthermore, we can gain information on *r*_init_ and *v*_init_ of the CME by ELEvoHI. In this model, *v*_init_ is derived from HI data after the conversion from elongation to distance. It depends on the chosen geometry of the CME front shape (within the ecliptic) and on the starting point of DBMF within the model. Figure 8a shows the distribution of different values of *γ*, grouped in bins of a size of 0.05 × 10^−7^ km^−1^ and color coded based on Δ*t*. Surprisingly, all of the exact predictions (within ±0.5 hr) and almost all predictions within ±2 hr have a *γ* of 0.15 or 0.2 × 10^−7^ km^−1^. Additionally, these values—along with *γ* = 0.25 × 10^−7^ km^−1^—are the most frequently resulting drag parameters in the whole ensemble. The same approach, but for *w*, *r*_init_, and *v*_init_, is presented in Figures 8b–8d. Here we find the same picture: the best predictions result from the most frequent values.

**Figure 9:**
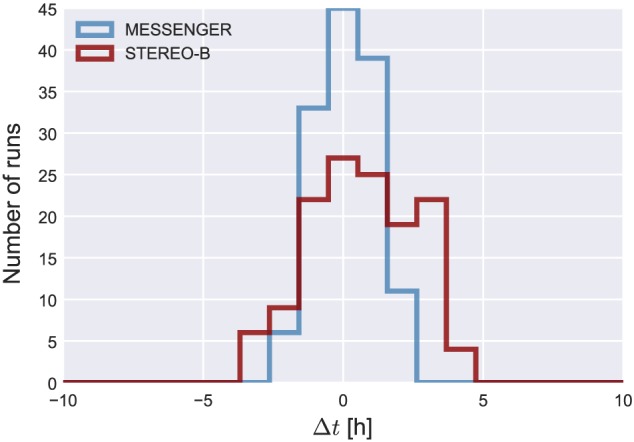
Frequency distribution of the predictions with w = 342 km/s and 0.15 × 10^−7^ km^−1^≤γ≤0.25 × 10^−7^ km^−1^, 29 R_⊙_≤r_init_≤37 R_⊙_, and 490 km/s ≤v_init_≤570 km/s, that is, the most frequent values as seen in Figure 8. STEREO = Solar TErrestrial RElations Observatory; MESSENGER = MErcury Surface, Space ENvironment, GEochemistry, and Ranging.

Since ELEvoHI is planned to be used as real‐time prediction tool as soon as STEREO‐A provides near‐real‐time observations from the Sun‐Earth line again, we try to find an approach to limit the ELEvoHI ensemble predictions, which can be used in real time; that is, it should be easy and fast. When ensemble modeling is performed, taking into account the frequency distribution of, for example, *γ* and *w* seems to be an easy and beneficial way to limit the ensemble results. As a proof of concept, we extract all runs where 0.15 × 10^−7^ km^−1^≤*γ*≤0.25 × 10^−7^ km^−1^ and *w* = 342 km/s, the runs with the most frequent values of *γ* and *w*. This is the case for 140 runs. Additional limitation using the frequency distributions of *r*_init_ and *v*_init_ by taking into account only those runs with 29 R_⊙_≤*r*_init_≤37 R_⊙_ and 490 km/s ≤*v*_init_≤570 km/s leads to a further reduction to 134 runs. Figure 9 shows the distribution of this sample and reveals that, indeed, the predictions can be improved. In detail, the mean Δ*t* at MESSENGER is 0.1 ± 1 hr (mean absolute error is 0.9 ± 0.6 hr), the mean Δ*t* at STEREO‐B is 0.7 ± 1.8 hr (mean absolute error is 1.6 ± 1.1 hr).

**Figure 10:**
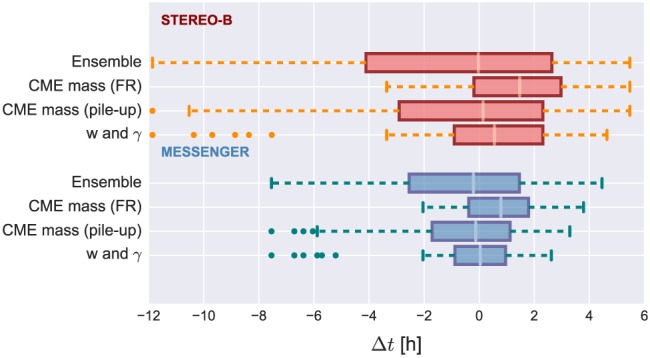
Comparison of the tested constraints to the ensemble results. Predictions for STEREO‐B are red; predictions for MESSENGER are blue. In each case, the upper box represents the whole ensemble, the second corresponds to the mass‐constrained sample based on the GCS flux rope fit (smaller angular width), and the third corresponds to the mass‐constrained sample based on the GCS fit to the pileup region (larger angular width). The lower boxes show the ensemble resulting from the constraint of the most frequent values of γ and w. The orange and green dots mark outliers. STEREO = Solar TErrestrial RElations Observatory; MESSENGER = MErcury Surface, Space ENvironment, GEochemistry, and Ranging; CME = coronal mass ejection; GCS = Graduated Cylindrical Shell.

Figure 10 presents a comparison of the two possibilities to limit the ELEvoHI ensemble prediction. The red boxes correspond to predictions for STEREO‐B; the blue boxes correspond to predictions for MESSENGER. In each case, the upper box represents the whole ensemble, while the two middle boxes stand for the mass‐constrained sample and the lower box corresponds to the constraint using the most frequent values of *γ* and *w*. The latter method is fast and simple, because no mass derivation is needed and the two parameters and their distribution directly result from the ensemble prediction. Furthermore, it is also more accurate than the mass constraint and can easily be used in real time. The outliers (orange dots) can be excluded by further limiting the ensemble using the initial distance and speed distributions.

### 5.3 Comparison of the ELEvoHI CME Front Shape to WSA‐ENLIL+Cone Model

Usually, the WSA‐ENLIL+Cone model is used to predict CME arrivals. However, we are interested in the evolution of the CME front shape and interaction with the background solar wind, and therefore, the input parameters were varied until the best matches with the in situ arrival times are found. Thus, the WSA‐ENLIL+Cone model was not used to perform an arrival prediction. The resulting CME front shape from this model is compared to the rigid shape assumed by ELEvoHI. The global 3‐D MHD ENLIL model provides a time‐dependent description of the background solar wind plasma and magnetic field using the WSA coronal model (Arge & Pizzo, 2000; Arge et al., 2004) as input at the inner boundary of 21.5 R_⊙_ (Odstrčil, 2003; Odstrčil & Pizzo, 1999a, 1999b; Odstrčil et al., 2004, 1996). A homogeneous, overpressured hydrodynamic plasma cloud is launched through the inner boundary of the heliospheric computational domain and into the background solar wind. WSA coronal maps provide the magnetic field and solar wind speed at the boundary between the coronal and heliospheric models at 21.5 R_⊙_. ENLIL version 2.8 was used in this work, with a time‐dependent inner boundary constructed from a series of daily input WSA synoptic maps, each computed from a new Global Oscillation Network Group (Harvey et al., 1996) daily synoptic *QuickReduce* magnetogram every 24 hr at the ENLIL inner boundary. For this study the WSA‐ENLIL+Cone simulations have a 1° spatial resolution (high) and spherical grid size of 1,536 × 120 × 360 (*r*,*θ*,*ϕ*) with a 3‐hr 3‐D output cadence and 5 min output cadence at locations of interest. The simulation range was 0.1 to 2.1 AU in radius, *r*, −60° to +60° in latitude, *θ*, and 0° to 360° in longitude, *ϕ*.

**Figure 11:**
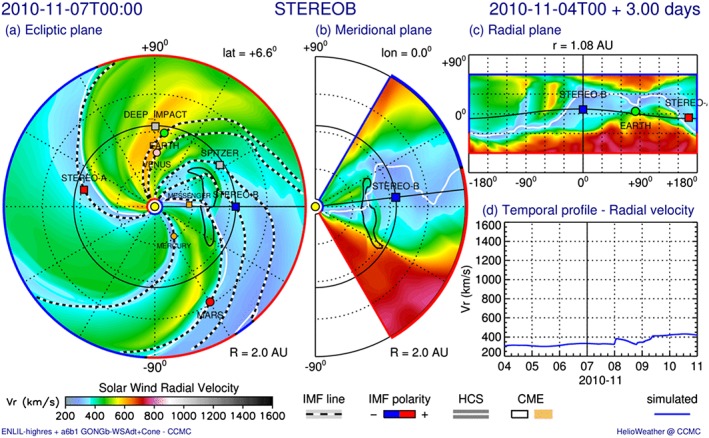
Velocity contour plot of the CME simulation in the (a) ecliptic plane, (b) meridional plane of STEREO‐B, and (c) 1‐AU sphere in cylindrical projection on 7 November 2010 at 00:00 UT. Panel (d) shows the simulated (blue) radial velocity profile at STEREO‐B. STEREO = Solar TErrestrial RElations Observatory; MESSENGER = MErcury Surface, Space ENvironment, GEochemistry, and Ranging; CME‐coronal mass ejection; IMF = interplanetary magnetic field; HCS = heliospheric current sheet.

Figure 11 shows a velocity contour plot of the simulated CME in the ecliptic plane (a), the meridional plane of STEREO‐B (b), a 1‐AU sphere in cylindrical projection (c), and the simulated in situ solar wind speed at STEREO‐B (d). The figure shows that nearly the center part of the CME impacts STEREO‐B, followed by a high‐speed stream as seen in Figure 2. From the ENLIL run it can be seen how the simulated CME deforms during propagation. At its onset, the shape is almost elliptical, that is, in agreement with ELEvoHI, while during evolution a concave shape develops at the portion heading toward STEREO‐B. One reason for this deformation may be the different drag regime on the other side of the heliospheric current sheet (white line) because of the higher solar wind speed (500 compared to 300 km/s). Interestingly, this dip in the CME front could lead to an arrival time difference of 15 hr compared to the arrival of a uniform shape. Here the question arises if it is even possible to reduce today's real‐time prediction error to less than half a day. Nevertheless, it is worth noting that fast and massive CMEs, which are expected to cause the largest space weather effects, are less likely distorted by other structures in the ambient medium.

## 6 Summary and Discussion

ELEvoHI is designed to predict CME arrivals in real time provided that HI data are available in (near) real time. To reach this goal until STEREO‐A is close enough at the Sun‐Earth line providing data, which can be used to predict Earth‐directed CMEs, we assess and further develop this tool using science data. To predict CME arrivals with a high degree of accuracy, it is of high importance to have suitable input parameters available. In this study, we examined in which way GCS fitting to coronagraph observations is able to provide information on the shape of the CME front within the ecliptic plane and which influence the three shape‐related input parameters (propagation direction, angular width, and curvature of the front) have on the prediction result. Two different GCS fits were performed, namely, one to the CME flux rope (as commonly done) and one capturing the dense area surrounding the flux rope. This latter GCS fit is assumed to be more consistent with other assumptions of ELEvoHI, especially with HI elongation measurements, which are taken at the shock front of the CME and not at the CME cavity. In order to identify the shape of the CME within the ecliptic, the GCS shape was subtended with the ecliptic plane resulting in an ellipse‐shaped CME front. From this ellipse the needed input parameters and their error ranges were measured. Within this range of input parameters, an ensemble of 339 ELEvoHI runs was performed, predicting the arrival times and speeds at MESSENGER and STEREO‐B. The ensemble mean for predictions at MESSENGER was Δ*t* =− 0.6 ± 2.7 hr and for STEREO‐B Δ*t* =− 0.9 ± 4.2 hr. This is an impressive result, but one should keep in mind that case studies always lead to better predictions than studies dealing with larger event samples. Furthermore, HI science data are not available in real time but were used in this study. If using HI beacon data leads to similar results needs to be further investigated in future studies.

### 6.1 L1 Point as Potential Location for an HI Observer

In this paper the HI observations used in the arrival time predictions were obtained from the same spacecraft that detected the CME in situ; that is, the CME appeared as a halo CME. Our study shows that—at least for this one event—halo CME observations from HI can lead to a feasible arrival prediction. Of course, a study with a large event sample is needed to investigate the optimal location for HI observations.

DeForest et al. (2016) and Harrison et al. (2017) already pointed out that the observation of Earth‐directed CMEs may also be possible from the L1 point or in low Earth orbit, the latter location was already proposed and simulated by DeForest and Howard (2015). An operational space weather mission at L1 or low Earth orbit instead of L4 or L5 would reduce the costs of such a mission by a noteworthy amount. However, an L5 observatory has more benefits than only providing HI observations from the side of Earth‐directed CMEs, for example, the observation of active regions before they rotate onto the Earth‐facing side of the Sun. Therefore, the most efficient way to improve space weather prediction would be to have both an HI observatory at L5 and L1.

### 6.2 Usage of ELEvoHI Ensemble Predictions

In this study, we found a possibility to constrain the ELEvoHI ensemble prediction in a way that is easy and fast to conduct and leads to promising results for real‐time predictions. Two different procedures were tested. The first approach is an exclusion of runs for which the mass resulting from ELEvoHI was not in agreement with the mass calculated from coronagraph observations. This approach is an additional verification of *γ* resulting from ELEvoHI to avoid unphysical results. We calculated two different values for the cross‐section area, on which the drag force is acting on, based on the angular width resulting from the GCS fitting to the CME flux rope and to the dense region preceding the flux rope, respectively. The cross‐section area is needed to derive the mass from the ELEvoHI output.

We found that mass calculated from the angular width derived from the GCS fit to the CME flux rope reveals a better constraint of the ensemble results than the mass calculated from the wider GCS fit to the CME shock. Limiting the ensemble runs to those having the same mass (±50%) calculated from ELEvoHI as from coronagraph observations results in an error range of almost 50% less (±2 hr) than for the whole ensemble when using the more narrow GCS fit. All in all, we find a good agreement between the CME mass derived from observations and the ELEvoHI mass, which can be seen as a verification of the drag‐parameter resulting from DBMF within ELEvoHI. However, the usage of the CME mass to confine the ELEvoHI ensemble prediction might be too defective and too slow for real‐time predictions.

For the second approach, the frequency distributions of *γ*, *w*, *r*_init_, and *v*_init_ resulting from the ensemble run (339 runs) showed that the most accurate predictions are connected to their most frequent values, resulting from drag‐based fitting implemented within ELEvoHI. Taking into account only those runs where *γ* as well as *w*, *r*_init_ and *v*_init_ belong to the most prevalent values, we were able to further constrain the ensemble prediction at MESSENGER to a mean error of Δ*t* = 0.1 ± 1 hr and at STEREO‐B to Δ*t* = 0.7 ± 1.8 hr and to a mean absolute error of Δ*t* = 0.9 ± 0.6 hr at MESSENGER and to Δ*t* = 1.6 ± 1.1 hr at STEREO‐B.

Ensemble forecasting seems to be a good possibility to use ELEvoHI for real‐time prediction. A test by applying ELEvoHI to a large sample is going to reveal if this method is indeed an improvement or not. Furthermore, it may be worth testing if a GCS fit in advance of the ELEvoHI run can be avoided when doing an ensemble prediction. Varying the input parameters within their common values, for example, 35° ≤*λ*≤85° and 0.4≤*f*≤1, and extracting from the ensemble results the runs with the most frequent values of *γ*, *w*, *r*_init_, and *v*_init_ could speed up the prediction and make the usage of additional GCS fitting redundant.

## 7 Data Sources

**Image data**

STEREO/HI: https://www.ukssdc.ac.uk/solar/stereo/data.html

STEREO/COR2: https://sharpp.nrl.navy.mil/postflight/lz/L0/a/img/cor2/ and https://sharpp.nrl.navy.mil/postflight/lz/L0/b/img/cor2/

SoHO/LASCO: https://sharpp.nrl.navy.mil/cgi-bin/swdbi/lasco/img_short/form

**In situ data**

STEREO: http://aten.igpp.ucla.edu/forms/stereo/level2_plasma_and_magnetic_field.html

MESSENGER: https://pds-ppi.igpp.ucla.edu

Spitzer Space Telescope: https://doi.org/10.6084/m9.figshare.5752848.v3

**Model results**

ENLIL‐WSA+Cone simulation results are provided by the Community Coordinated Modeling Center through https://ccmc.gsfc.nasa.gov/database_SH/Tanja_Amerstorfer_032118_SH_2.php

ELEvoHI model results, the HI time‐elongation measurements, the animated version of Figure 5 are available under https://doi.org/10.6084/m9.figshare.5752848.v3.

## Supporting information



Supporting Information S1

Movie S1
