# Supplementary material for: Ensemble Prediction of a Halo Coronal Mass Ejection Using Heliospheric Imagers
Source: Space Weather. 2018 Jul 2;16(7):784–801. doi: 10.1029/2017SW001786 (PMC6099306; doi:10.1029/2017SW001786)
Supplement: Supplementary file 1 — Supporting Information S1 [file SWE-16-784-s001.docx]

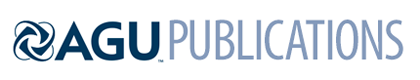


*Space Weather*

Supporting Information for

**Ensemble Prediction of a Halo Coronal Mass Ejection Using Heliospheric Imagers**

T. Amerstorfer1, C. Möstl1, P. Hess2, M. Temmer3, M.L. Mays4, M.A. Reiss1, P. Lowrance5, Ph.-A. Bourdin1

1Space Research Institute, Austrian Academy of Sciences, 8042 Graz, Austria

2NRC Research Associate, U.S. Naval Research Laboratory Washington, DC 20375, USA

3Institute of Physics, University of Graz, 8010 Graz, Austria

4Heliophysics Science Division, NASA Goddard Space Flight Center, Greenbelt, MD 20771, USA

5IPAC, MS 314-6, California Institute of Technology, 1200 E. California Blvd, Pasadena, CA 91125, USA

**Additional Supporting Information (Files uploaded separately)**

Captions for Movies S1

**Introduction**

The file ms01.mp4 shows a visualization of the ELEvoHI ensemble prediction for the coronal mass ejection from November 3 2010. To create this movie, information on the shock arrival time and magnetic field strength from in situ data at MESSENGER and STEREO-B are used as well as elongation measurements extracted from STEREO-B heliospheric imager observations.

Movie S1. Animated visualization of ELEvoHI ensemble run. The black curves correspond to the CME shapes leading to the best prediction at MESSENGER and STEREO-B, respectively. The dark gray area is the entity of all other runs from the ensemble. The blue tangent, as long as it is solid, corresponds to the first and last elongation measurement used for the prediction. These elongation angles correspond to the times, when the CME apex is between 30 and 100 R⊙, i.e. the start and end times of the fit. When the blue tangent changes to a dashed linestyle, the corresponding HI elongations show the consistency with the prediction, but these observations are not taken into account for the predictions anymore. The last position of the dashed line marks the last HI observation from this CME available. The size of the filled circles at the location of MESSENGER and STEREO-B mark the arrival times and magnetic field strengths measured in situ.
